# Minimally Invasive and Endoscopic Approach to Esophageal Perforation

**DOI:** 10.5152/eurasianjmed.2022.21135

**Published:** 2022-02-01

**Authors:** Atilla Eroglu, Yener Aydin, Ali Bilal Ulas

**Affiliations:** Department of Thoracic Surgery, Atatürk University School of Medicine, Erzurum, Turkey

**Keywords:** Endoscopic management, esophageal leak, esophageal perforation, esophageal stent, minimally invasive surgery

## Abstract

Although esophageal perforation is rare, it is a life-threatening condition. The esophagus is very sensitive to rupture and perforation due to the absence of a serosal layer. More than half of the esophageal perforations are iatrogenic and most occur during endoscopy. Around 55% of esophageal injuries occur in the intrathoracic region, 25% in the cervical region, and 20% in the abdominal region. Clinical manifestations and mortality are related to various components such as the etiology, localization, type of injury, severity of contamination, injury to adjacent mediastinal structures, and time from perforation to treatment. When perforation occurs in the esophagus, saliva, stomach contents, bile fluid, and other secretions may pass into the mediastinum causing mediastinal emphysema, inflammation followed by mediastinal necrosis, and chemical mediastinitis. Early clinical suspicion and imaging are essential for successful treatment. Despite advances in technology and treatment, the morbidity and mortality rate due to esophageal perforation is still higher than 20% according to the reported studies. Until now, the main treatment of esophageal perforation was the surgical approach. Nowadays, endoluminal procedures such as endoscopic vacuum therapy, endoscopic stent placement, endoscopic clip closure, endoscopic suturing, and tissue adhesives have started to be applied more. In this review, the minimally invasive and endoscopic approach methods suitable for esophageal perforation according to the characteristics of the patients and the structure of the perforation were examined.

## Introduction

The esophagus is a structure exposed to serious injuries located in the neck, mediastinum, and abdomen, which is approximately 25 cm long. This structure, through which food is transmitted from the oropharynx to the stomach, is adjacent to vital organs. The esophagus does not have a serosa layer. It is an organ that can be easily injured and has a low possibility of end-to-end anastomosis due to its thin wall and weak blood supply. An abnormal connection of the esophagus with the pleural cavity, mediastinum, or peritoneal cavity is referred to as esophageal rupture or perforation.^1^

Around 55% of esophageal injuries occur in the intrathoracic region, 25% in the cervical region, and 20% in the abdominal region.^1^ Esophageal perforation is a matter of controversy in diagnosis and treatment due to its rare occurrence and variability in its occurrence. Until now, the main treatment of esophageal perforation was the surgical approach. Nowadays, endoluminal procedures such as endoscopic vacuum therapy, endoscopic stent placement, endoscopic clip closure, endoscopic suturing, and tissue adhesives have started to be applied more.^2^ In this review, the minimally invasive approach methods suitable for esophageal perforation according to the characteristics of the patients and the structure of the perforation were examined.

### Etiology and Incidence

More than half of the esophageal perforations are iatrogenic, most of which occur while performing endoscopy. The perforation risk is low in diagnostic endoscopy performed by flexible endoscopy. However, therapeutic interventions such as foreign body removal, stent placement, pneumatic dilatation, hemostasis, endoscopic ablation techniques, and cancer palliation can significantly increase the risk of perforation.^1,2^ While the esophageal damage risk is 0.18-0.03% in flexible endoscopy, this rate is 0.11% in rigid esophagoscopy; when therapeutic interventions are added, the rate can rise to 10-15%.^3^ Esophageal perforation occurs spontaneously in approximately 15% of the cases.^1,4^ Esophageal foreign bodies take third place in perforation etiology. Esophageal perforation may occur less frequently due to penetrating or blunt trauma, intraoperative injury, and malignancy.^2,3^

### Clinical Findings

Physical examination findings and symptoms vary according to the reason, location, and time of the injury. Clinical findings in esophageal perforations are unclear at first and usually become apparent after 24 hours. In the first hours of the injury, if there are no concomitant complications such as subcutaneous emphysema or pneumothorax, pathology may not be detected on physical examination. This seems more common in patients with iatrogenic perforation and those who have not been fed orally. The patient may present with sepsis symptoms hours or days after the perforation due to oral intake of food.^5,6^

The most common symptoms are dysphagia, dyspnea, pain, fever, and subcutaneous emphysema. Pain is the most common but completely non-specific symptom. Although fever is not specific, it is a sign of the possible infection onset and systemic inflammatory response. Pain, odynophagia, and dysphagia in the cervical region are common. The patient often has hematemesis. On examination, neck swelling, subcutaneous crepitation, and pain in deep palpation can be detected. Neck stiffness may occur in flexion and extension, and pain may occur after a few hours. In the perforation of the thoracic esophagus, substernal and interscapular pain, fever, and dysphagia are seen in the early period. The patient usually describes the dysphagia close to the perforation site. The severity of dyspnoea varies according to the degree of pleural contamination, the amount of hydropneumothorax, and the presence of airway compression. There may be tenderness in the upper abdomen. At auscultation, crepitation of the mediastinal air can be heard.^5,6^

### Diagnosis

Even nonspecific minimal symptoms should not be overlooked in the diagnosis in penetrating and blunt traumas of the neck and chest, ingestion of a caustic substance, ingestion of foreign body or foreign body removal from the esophagus, in esophageal interventions or interventions through the esophagus, and in patients who have recently undergone surgery.

Chest x-ray radiography is used to discover air originating from the perforation. Subcutaneous emphysema is seen in cervical esophageal injury, and mediastinal emphysema is seen in thoracic esophageal rupture. Under the diaphragm, free air is a clear marker in abdominal esophageal perforation. Furthermore, some findings that can be seen on chest x-ray radiography include hydropneumothorax, hydrothorax, pleural effusion, the air in the soft tissues of the prevertebral space, and pericardial effusion.^2-4^

Esophagography is required in all esophageal perforation cases to support the diagnosis, to locate the perforation, and to plan the treatment method. In cases with perforation in the lower esophageal part, it is often seen that the contrast medium overflows into the mediastinum or the pleural space. If doubts are still present after esophagography using a water-soluble contrast agent or if it cannot be anatomically precisely localized, the process can be repeated using barium.^2-4^

Oral contrast administration immediately before the computed tomography (CT) scan is alternative for diagnosis. Computed tomography scan can show mediastinal enlargement, pneumomediastinum, pneumothorax, subcutaneous emphysema, lesion level, abscess cavities, and foreign body if any. Sometimes CT can detect even little extravasations of contrast agents which are not visible on standard radiographs.^3,4,7^

The most definitive diagnosis of rupture is to be seen endoscopically. Owing to the esophagoscopy, both additional pathologies and the level of rupture are determined and the method to be selected is decided. However, it is controversial whether or not to perform an endoscopy in case of perforation suspicion. Little perforations can be overlooked by even experienced endoscopists. Besides, accessing the injured area by the endoscope can make the perforation even larger and lead to further contamination.^1,2,6^

## Selection and Goals of the Treatment Method

Successful in the treatment of perforation is related to multiple components such as the duration between rupture formation and the moment of diagnosis, the contamination degree, the size and localization of the rupture, and the general status of the patient. The most important component affecting the outcome of esophageal perforation is the time from the moment of perforation to diagnosis.^6,7^ There is uncertainty in the optimal treatment of perforation as there is no single strategy to adequately deal with most of these situations.

Prompt diagnosis, antibiotic therapy, appropriate hemodynamic monitoring and support, control of extraluminal contamination, and restoration of luminal integrity constitute the goals of treatment in a patient with esophageal perforation.^8,9^

### Surgery

Although many authors advocated repairing the perforation area with supportive tissue in the early period in all perforations, the primary repair was not recommended in cases with late admission. Many reviews and series have reported that treatment in the first 24 hours produces successful results.^10,11^ The operative approach to be chosen depends on the hemodynamic status, the convenience of the esophageal muscle and mucosa layers for primary repair, and the presence of other pathologies. These surgical methods are drainage alone, drainage and decortication, primary repair with or without tissue support, controlled fistula creation by T-tube, esophageal exclusion, or esophageal resection.^5,6,12^

Although many authors line up with the open surgical approach of esophageal perforations, some authors have also achieved excellent results by nonoperative treatment and percutaneous control of mediastinal sepsis.^2,5,6,13^ With the development of endoluminal and minimally invasive procedures, the usage of minimally invasive approaches in perforation treatment is increasing today.^14,15^

## Endoscopic Treatment

In the last 2 decades, a significant increase has been observed in the use of stent placement, endoscopic suturing, clip placement, tissue adhesives, and endoscopic vacuum therapy in the perforation treatment. Its feature of including diagnosis and treatment interventions increases the preference for endoscopy. These treatments are sometimes used alone, sometimes combined with laparoscopy or thoracoscopy. In the case of the hybrid approach, the perforation area is usually closed endoscopically, while the mediastinal and peritoneal drainage and debridement can be performed minimally invasively by the thoracoscopic or laparoscopic approach. The hybrid procedure is most common today in the form of primary repair or thoracoscopic drainage with endoscopic stent placement.^19-21^

### Esophageal Stent Placement

Esophageal stents are most commonly used in patients with esophageal cancer for whom surgery is not suitable. Also, the use of esophageal stents in the treatment of benign oesophageal strictures, esophageal perforation, benign-malignant esophago-respiratory fistulas, and postoperative anastomotic leaks has been increasing.^22,23^ The major advantages of stent placement are instant control of perforations, preservation of the esophageal wall during mucosal healing, prevention of stricture formation, and early oral feeding.^24-26^ Today, expandable stents are generally used, and the use of rigid stents is extremely rare. While the rate of surgical intervention has decreased in the esophageal perforation treatment in recent years, stent placement has become quite common.^2,3^

### Self-Expandable Metallic Stents

Self-expandable metallic stents (SEMS) are the first expandable stents designed. These are divided into 3 types: uncovered, partially covered, and fully covered. The classic example of uncoated stents is the Ultraflex (Boston Scientific, Marlborough, MA) stent. These stents are usually permanent. Attempts to remove the stent can cause serious morbidity. The fully covered stent consists of a membrane that covers the stent along its length. These are usually double funnel-shaped and have a circumferential suture-shaped suture at the ends. In the standard endoscope, the stent can be retracted by holding this suture with forceps. The coating is usually made of silicone or a polymer that prevents tissue ingrowth. The benefit of using the fully covered stent is that it rarely becomes embedded in the tissue and can be removed more easily in the future. It can also close defects such as perforation and isolate the injury area. In partially covered stents, the proximal and distal ends are not covered and the stent body is covered with a membrane. Partially covered stents have characteristics of both fully covered and uncovered stent types. The uncovered proximal and distal extended ends of a partially covered stent help complete stent placement and prevent migration. However, the stent body is covered and prevents tissue ingrowth into the stent.^27,28^

Closing the perforation with an esophageal stent is one of the most effective methods today. The placement of full covered SEMS in esophageal perforations has several important functions. The stent covers the perforated area and removes the esophageal contents along with this site. Thus, the resumption of oral nutrition is facilitated and contamination of the extraluminal structures is prevented. Another advantage of the stent is that it provides tissue re-epithelization. Fully covered stents are optimum for controlling leakage. However, these also have a higher probability of migration. It is very important to ensure proper drainage of the perforation area. Because the fully covered stent can prevent leakage through the wall of esophagus, but it may prevent the cavity from draining sufficiently and may also lead to sepsis.^2,29^

The location and length of the perforation should be demonstrated by contrast-enhanced esophagogram or endoscopy before stent placement. The patient can be relieved with sedatives and analgesics (intravenous 3-5 mg of midazolam and 50 mg of meperidine). Esophageal SEMS can be placed under the guidance of endoscopy, with or without fluoroscopy. Partial or fully covered SEMS is placed by centering the perforation area. The stent can be fixed to the esophagus with clips to prevent migration. There are ready-made stents with thread and the thread can be fixed to the nose wings or earlobe after the stent is placed. Stents are usually removed after 2-3 weeks of placement.^24-26^

### Self-Expandable Plastic Stents

The only self-expandable plastic stents (SEPS) available today are Polyflex stents (Boston Scientific). It is made of a polyester mesh with an inner silicone liner designed to reduce stent tissue reaction. While the middle and lower parts of the stent are of the same caliber, the upper part of the stent is enlarged to reduce stent migration. The stent is first loaded into the delivery system by the surgeon. This delivery system is quite bulky (12-14 mm) and requires stenosis dilatation before stent placement in narrow structures. The same stent can be removed after migration and reloaded into the delivery device and reused.^30,31^

The self-expandable plastic stents have high efficiency in the control of esophageal leaks and perforations. There are some benefits over metal stents in esophageal perforation control. The soft material provides a safe and effective force to seal the leak, and the silicone membrane prevents tissue ingrowth. This allows easy repositioning and removal of the stent. However, SEPS placement is more difficult and the rate of migration is higher.^2,32^ Kamarajah et al^33^ reported the technical and clinical success rates of metallic and plastic stents in postoperative anastomotic leaks, and spontaneous and iatrogenic oesophageal perforations as 96% and 87%, respectively, in their recent review of 1752 cases. Plastic stents had higher rates of migration and repositioning and lower technical success than metallic stents. In cases with perforation, plastic stents were related with significantly lower technical success.

### Endoscopic Suturing

Endoscopic sutures can be used for fixation to prevent migration of esophageal stents, as well as for primary repair of esophageal perforations. Endoscopic suturing is used for both acute perforations and chronic fistulas. The endoscopic suture technique allows the closure of large defects that cannot be closed with other endoscopic interventions by full-thickness suturing. The device requires a dual-channel therapeutic endoscope. Tissue approximation and suture placement can be facilitated by holding forceps that retract the tissue. Additional auxiliary parts can be inserted through the working channel of the endoscope. The OverStitch endoscopic suturing system allows interrupted or continuous stitches without the need to remove the device.^34,35^

One possible complication of using suture devices is mucosal damage. However, this risk can be quite avoided by placing an overtube before the procedure. Unlike other endoscopic techniques for closing esophageal perforations, endoscopic suturing requires a learning curve for more widespread use.^4^

### Tissue Adhesives and Glue

Fibrin glue and cyanoacrylate have been used successfully in the treatment of surgical anastomotic leaks and small diameter fistulas. These sealants can be used alone or in conjunction with other endoscopic interventions. Fibrin glue is applied via a double lumen catheter after the cleansing of secretions to dry the targeted area to form a fibrin clot. The underlying epithelium must be peeled off with a cytology brush before fibrin glue is applied.^4,36^

Cyanoacrylate can be applied to the infected area as it has antibacterial properties. Cyanoacrylate has a stronger adhesion than fibrin glue. Cyanoacrylate is resistant to the stomach or pancreatic enzymes and ensures the successful closure of fistulas. Moreover, fibrin is difficult to use as it performs better in dry areas. Fibrin-based sealant prevents the passage of gastrointestinal contents through the fistula and supports tissue repair. Despite the high success rate, a large perforation is unlikely to be successfully closed with tissue adhesives alone.^36,37^

### Endoscopic Clip

Clip closure is one of the most commonly used endoscopic methods for closing gastrointestinal perforations. In some cases, clips may be used with stents. However, apart from preventing migration of stents, it can be used alone for the primary closure of perforation. Small iatrogenic perforations that are instantly diagnosed are candidates for using the endoscopic clip closure method. There are 2 types of endoscopic clip applications: through-the-scope clip (TTSC) and over-the-scope clip (OTSC).^2,4,9,38^

While TTSC was originally designed for hemostasis, it was later developed to be used to close iatrogenic perforations. Through-the-scope clip can be used to cover perforations smaller than 2 cm, provided that the tissue surrounding the edges is viable and applicable. Clip application may be difficult if the tissue around the defect is inflamed or hardened. It is recommended to continue closing proximally starting from the distal part of the perforation. When the edges of the perforated esophagus are brought close enough, the clip is placed over the grasped tissue.^9,34,39^

Over-the-scope clip is another method for closing the perforation. The clips come in 3 sizes: 11 mm, 12 mm, and 14 mm. The clip is preloaded on a transparent cover mounted at the end of the endoscope. Before the clip is placed, the defect is pulled with forceps and the clip is placed more effectively. Unlike TTSCs, OTSCs have a greater closing force. However, OTSC insertion may fail for defects greater than 20 mm or for inflammatory or necrotic conditions.^36,40-42^

### Endoscopic vacuum therapy (EVT) or EndoSponge

Following perforation, endoscopic drainage of the mediastinal or peritoneal collection or abscess can be achieved by endoscopic vacuum therapy (EVT) . The device has parts of a sponge and a tube attached to an external vacuum absorber. This leads to a gentle, continuous suction on the tissue that clears secretions and induces the formation of granulation tissue. The endo-sponge can be inserted into the cavity or in the lumen and completely cover the perforation site. In the case of larger space, more than one sponge can be used.^34,43,44^

After the perforation area is determined, the cavity is irrigated and endoscopically debrided. Then endo-sponge is chosen depending on the size of the injury. A nasogastric tube is inserted through the nose and pulled through the mouth to insert the endo-sponge. The endo-sponge is then fixed to the tube using a strong permanent suture. An endoscopic grasper is then used to place the endo-sponge in the desired position. After proper localization is verified, negative pressure is started. The endo-sponge usually needs to be changed often, in 3-5 days. Endoscopic removal should be done by endoscopic vision after the leak has been covered.^36,45^

In a prospective cohort study, the results of closing upper gastrointestinal defects with EVT were evaluated in 52 cases. Recovery was observed in 94% of the cases without the need for surgical revision. Endoscopic vacuum therapy failed in three patients. Two of these patients died of bleeding from EVT. Post-intervention stenosis was observed in 4 cases during follow-up.^46^ Our treatment algorithm for esophageal perforation is presented in the figure.

## Conclusion

Esophageal perforation requires rapid diagnosis and treatment due to high morbidity and mortality. Advances in devices and techniques for endoscopic closure are hopeful for less invasive and easier treatment of esophageal perforations. However, the decision of surgical, endoscopic, conservative treatment should be individualized and evaluated by a multidisciplinary team. In some cases, hybrid methods combining endoscopic methods and thoracoscopy or laparoscopy may be required.

## Figures and Tables

**Figure 1.**
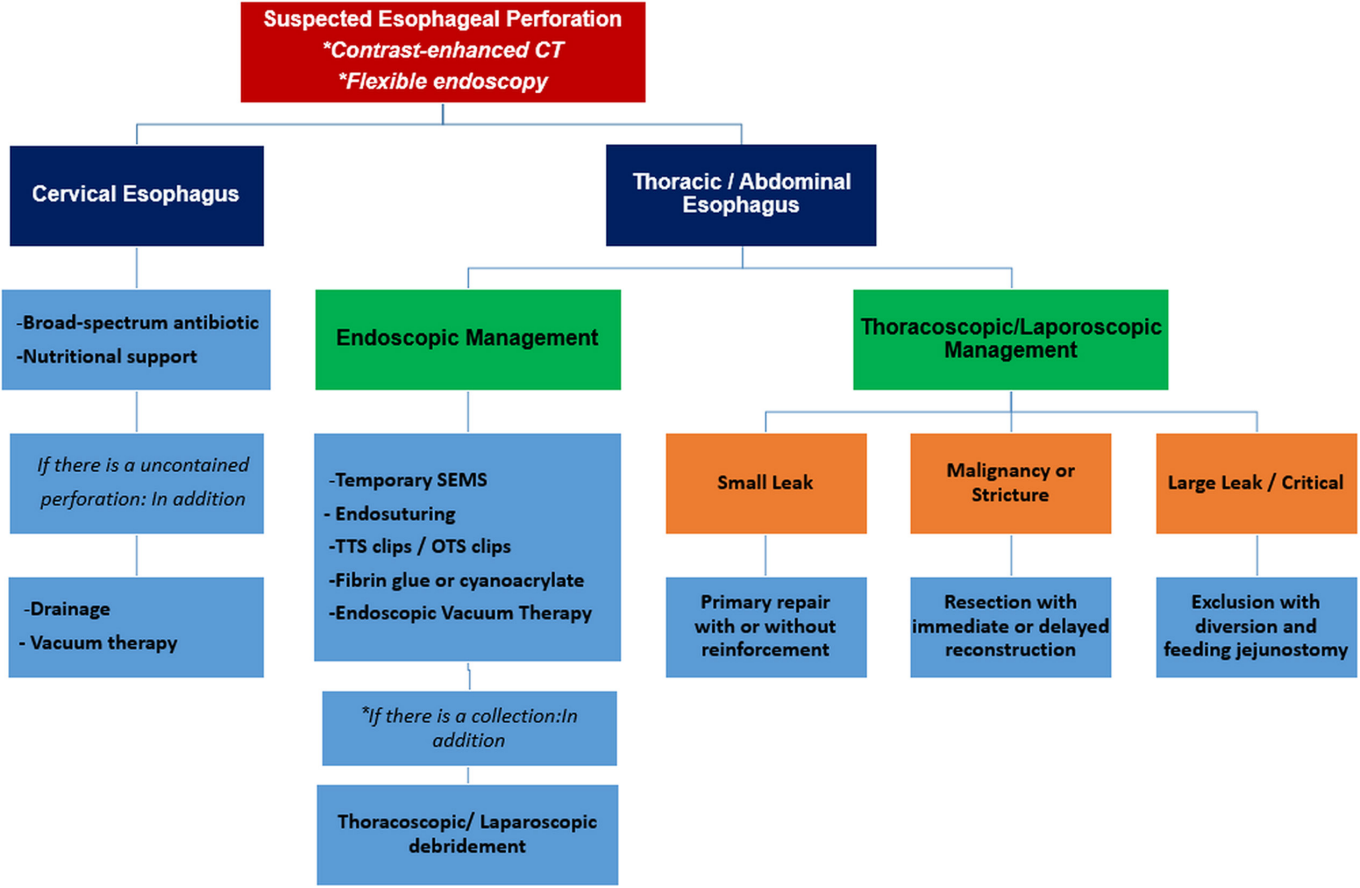
The treatment algorithm in esophageal perforation.
